# Data Resource Profile: The UK Cystic Fibrosis Registry

**DOI:** 10.1093/ije/dyx196

**Published:** 2017-10-03

**Authors:** David Taylor-Robinson, Olia Archangelidi, Siobhán B Carr, Rebecca Cosgriff, Elaine Gunn, Ruth H Keogh, Amy MacDougall, Simon Newsome, Daniela K Schlüter, Sanja Stanojevic, Diana Bilton, the CF-EpinNet collaboration

**Affiliations:** 1 Department of Public Health and Policy, University of Liverpool, Liverpool, UK; 2 National Heart and Lung Institute, Imperial College London, London, UK; 3 Department of Paediatric Respiratory Medicine, Royal Brompton and Harefield NHS Foundation Trust, London; 4 Cystic Fibrosis Trust, Aldgate, London, UK; 5 Department of Medical Statistics, London School of Hygiene and Tropical Medicine, London, UK; 6 Centre for Health Informatics, Computing and Statistics (CHICAS), Lancaster University, Lancaster, UK; 7 Translational Medicine, Hospital for Sick Children and Institute of Health Policy Management and Evaluation, University of Toronto, Toronto, ON, Canada

## Data resource basics

The UK Cystic Fibrosis Registry is a national, secure, centralized database sponsored and managed by the Cystic Fibrosis Trust, with UK National Health Service (NHS) research ethics approval and consent from each person for whom data are collected. First established in 1995, it records longitudinal health data on all people with cystic fibrosis (CF) in England, Wales, Scotland and Northern Ireland, and to date has captured data on over 12 000 individuals.

Cystic fibrosis is an inherited, chronic, progressive condition occurring in around 1 in 2500 live births in the UK, with around 200–300 new diagnoses annually. Children are generally diagnosed in the first few months of life with universal newborn screening being implemented in 2007 in the UK, though some people are diagnosed into adulthood. For instance, 29 people aged over 16 years were diagnosed with CF in the UK in 2015.^1^ Patients diagnosed with CF subsequently require intensive support from family and health care services. Most patients die prematurely from their disease through respiratory failure, and in the 1930 s and 40 s survival beyond childhood was rare.^2^ There have been impressive improvements in survival over subsequent decades; for instance, the median life expectancy of children with cystic fibrosis born in 1990 was estimated to be 40 years, double that of estimates 20 years earlier.^3^

In the UK, children with CF are treated in one of 33 specialist centres (associated with over 100 smaller network clinics). At between 16 years and 18 years of age, children transfer to one of 27 adult specialist centres. All centres and network clinics routinely collect data in a standardized fashion. When patients with CF attend a new CF centre in the UK, they or their parents consent to information on their health and treatment being collected and stored in the CF Registry. The patient information and consent form also covers the issue of linking registry data to the UK Office for National Statistics. When transitioning to adult services, the young adult is given the opportunity to confirm or withdraw consent. People with CF will also re-consent if they change their primary centre of care. The Registry records information about the health and treatment, and health care use (e.g. hospital days) and outcomes of patients from diagnosis onwards. The Registry contains personal identifiers, seen only by the local centre team and CF Registry data managers. Reports generated by the Registry are used as the evidence base for commissioning care and pharmacovigilance of new therapies. Harnessing the rich data in CF registries in the UK and beyond offers the opportunity to improve the lives of patients with CF now, and to establish an essential data resource for future research.

### History of the registry

The UK CF Registry started as the UK CF Database, which was established at the University of Dundee, Scotland, in 1995. Initially data were collected from 56 paediatric and adult CF clinics, using standardized forms, and validated through a system of double data entry, range checking and error correction.^4^ Between 2005 and 2007, the Cystic Fibrosis Trust rolled out a national UK-wide web-based system, following new ethics approval and re-consent of all patients, with migration of the data to a new system. The data collection system thus changed from a paper-based return system to using the online ‘PortCF’ software which mimicked the data collection and storage system used by the Cystic Fibrosis Foundation Patient Registry in the USA. During this transfer there was extensive retrospective data cleaning and checking. In 2012. the Registry commenced production of reports that are used by the NHS England to make payment by results (PbR) tariff payments to CF centres. Linking the Registry to NHS reimbursement processes significantly improved the completion of data. Recently, the PortCF system has been replaced by new UK CF Registry software developed by the UK-based web development specialists Net Solving Ltd. The new system has been developed with patient involvement and includes interactive elements that may, in due course, allow patients to access their own data. The UK CF Registry has thus far led to the production of 10 annual reports, with the latest 2015 data published in August 2016.^1^ The UK CF Registry Steering Committee was established in 2007 to oversee development of the Registry, annual reports and research governance; it meets regularly and includes medical, sponsor (Cystic Fibrosis Trust), commissioner, statistician, patient and parent representation. It has recently set up a sub-committee, the UKCF Registry Research Committee, to allow more detailed oversight and governance of this area.

## Data collected

The dataset contains: time-invariant variables, such as sex, genotype and date of birth; and longitudinal variables that change over time, such as weight and measures of lung function. CF patients are seen in the outpatient clinic for a comprehensive annual review, including evaluation of clinical status, pulmonary function, microbiology of respiratory tract secretions and use of major CF-related therapies. The minimum data collection requirement for the UK CF Registry is an annual dataset, usually taken from the annual review clinic visit. The data collected at the annual review are indicated in the dataset, and can be distinguished from ‘encounter’-based data collected in the interval between clinic visits. In this data resource profile, we only describe the annual review data which are the basis of the CF-Epinet dataset described below. Some clinics use the Registry to collect encounter data, but these are not systematically collected. Thus the data in the registry mostly derive from the annual review, rather than being encounter-based, though the annual review data include certain summaries of information since the previous review–for example. the number of days a patient has been on intravenous antibiotics and the best % forced epiratory volume in 1 s (FEV_1_) recorded in the previous year. Data are collected in key areasincluding: demographics (including genotyping and diagnosis data), hospital admissions and intravenous therapies, pulmonary function, chronic medications, culture and microbiology, health complications, nutritional assessment, physiotherapy, smoking, socioeconomic status and outcomes (death and transplants).^4^ Templates of the data collection forms showing all variables collected are available to download from the CF Registry portal as well as from the Cystic Fibrosis Trust Registry web page. ^5^

## Coverage

**Figure 1:**
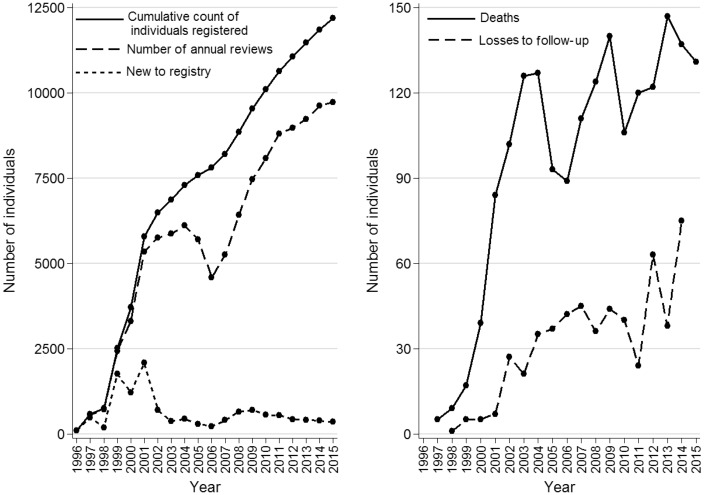
Cumulative count of individuals captured in the registry and number with annual review data in each year (left panel). Count of deaths and losses to follow-up (right panel).

Of the 22 countries providing data to the wider European Cystic Fibrosis Society Patient Registry,^6^ the UK CF Registry is the largest national database and the most complete in terms of coverage. Currently data on 12 201 patients are captured in the UK Registry (alive, dead or lost to follow-up) with 9734 (79.8%) still in follow-up at the end of 2015. In total there are data on over 100 000 annual assessments. Figure 1 shows the cumulative count of patients captured in the Registry by year, including patients who have died. Figure 1 also shows annual counts of annual reviews, deaths, and losses to follow-up (defined as patients with no annual reviews for 2 years in succession). The number of people captured in the dataset has increased year on year, with increases coinciding with the move to the web-based system, followed by the incentivizing of data collection for NHS funding purposes in England and Scotland in 2012. The number of patients for whom a ‘complete’ dataset, defined as the data required to produce the range of key clinical outcomes relating to growth, lung function and treatment presented in the annual reports, was recorded at 82% in 2009, and this has increased year on year, with the figure up to 89% for the latest (2015) annual report.^1^

**Figure 2:**
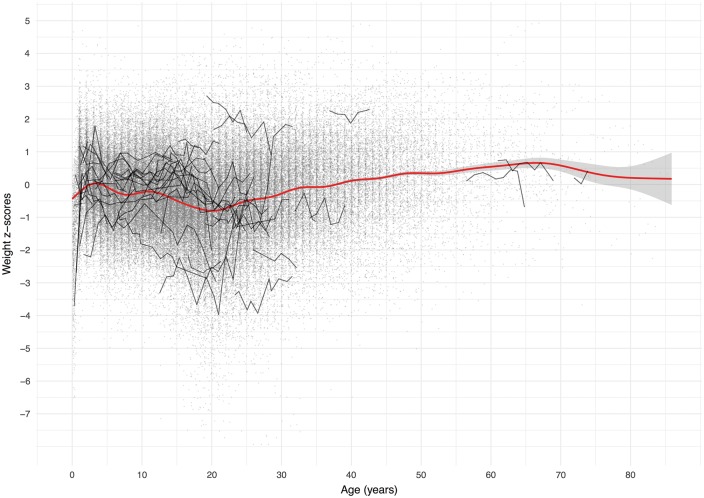
Spaghetti plot for weight z-score versus age, illustrating the longitudinal nature of the data collected in the UKCF Registry. Each dot (*n* = 117 482) represents a weight z-score measure on a person in the dataset. The smoothed cross-sectional population average is shown in red (95% confidence intervals) and 50 randomly selected individual trajectories are in black.

For the purposes of illustrating the longitudinal structure of the data, we consider the patient’s weight, since weight is one of the most commonly collected outcomes in the dataset, collected at 117 482 annual reviews on 12 201 patients between 1996 up to 2015 in the UK. A total 0f 78% of individuals had five or more weight measurements, with a mean number of nine measurements. Figure 2 shows all patient’s weight data, presented as age-standardized z-scores^7^ plotted against age, with randomly selected individual trajectories highlighted. Z-scores in adults were calculated assuming the weight-for-age distribution at age 19.

## Quality

Data quality is assured through a number of mechanisms. For clinicians and others entering data into the Registry, up-to-date user guides are available on the UK CF Registry portal and contextual help text is available next to individual variables, to instruct users as to how to interpret the question and use the software. Training videos are also available to assist clinicians in entering and monitoring data from their own clinic. Software functionality encourages CF centres to monitor their own data on an ongoing basis. A quality dashboard gives users an on-demand, at a glance view of their data completeness as well as summaries of key clinical indicators such as lung function, with live benchmarks against national averages.

The Registry data collection software performs data validation checks at point of entry. These include: the enforcing of mandatory data; range checks for clinically valid values; ensuring that only valid characters are entered; and text and visual prompts to ensure that data (including dates) are not illogical or conflicting and to encourage completion of core data. The Cystic Fibrosis Trust also supports a Registry Annual Meeting in July each year. This event is free to attend for all CF centre employees who enter data onto the Registry, and is designed to showcase current registry research and offer training and best practice-sharing opportunities.

The Cystic Fibrosis Trust Registry team perform training and validation visits at CF centres, to ensure that users are aware of data entry guidelines and encouraged to improve the completeness and accuracy of their data. After the data entry deadline on the 31 January each year, Registry data managers and statisticians perform a variety of data cleaning checks, such as tracking of patient transition from paediatric to adult clinics. Where longitudinal analysis detects apparent inconsistencies that cannot be automatically cleaned, the Registry team liaise directly with CF centres to check and, where relevant, correct data.

## Data resource use

Registry studies have been crucial in informing our understanding of the epidemiology, changing demographics, outcomes and treatments in CF. Two examples of analyses of UK Registry data are provided in the text box.

In addition to epidemiological studies, the UK CF Registry team produce reports on long-term drug safety required by the European Medicines Agency (EMA). These reports contain anonymized, aggregated data and allow safety- and efficacy-monitoring of new therapies for cystic fibrosis. There is also a facility to support Registry-based clinical trials. For instance, the UK CF Registry is currently, via a specially designed study module, running a CF Registry-based clinical trial, the cystic fibrosis (CF) anti-staphylococcal antibiotic prophylaxis trial (CF START): a randomized registry trial to assess the safety and efficacy of flucloxacillin as a long-term prophylaxis agent for infants with CF.^8^

Making use of the Port-CF system, with similar variables collected in the UK and USA, Goss and colleagues compare CF outcomes and use of treatments between the two countries. Their cross-sectional analysis suggested that the USA does better in terms of lung function in children, and one hypothesis raised is that this may be due to more intensive treatment in the early years in the USA.^9^

Studies making use of data from the UK CF Registry are increasing,^5–11^^,^^15^^,^^16^ but relatively few have made use of the longitudinal nature of the data. One longitudinal study has assessed the impact of socioeconomic status on outcomes and treatment use in the UK population.^7^ More disadvantaged children with CF in the UK were found to have significantly worse growth and lung function, and were more likely to have chronic *P. aeruginosa* infection. There was evidence that in the NHS, clinicians in making decisions about treatments for children take deprivation as well as disease status into account, and this may mitigate some effects of social disadvantage. The study raises concerns about the provision of therapies such as DNase to people living in disadvantaged areas.

### The Cystic Fibrosis Epidemiological Network (CF-EpiNet)

In recognition of the potential to better harness data from registries to improve patient outcomes, the Cystic Fibrosis Trust have funded a Strategic Research Centre (CF-EpiNet) focused on CF data and epidemiology. CF-EpiNet is focused on: Registry enhancement; application of state-of-the art statistical modelling techniques to longitudinal data; and economic modelling. An important part of this project will involve data cleaning, harmonization of variables over time and the generation of a research-ready ‘CF-EpiNet’ dataset. Algorithms and code for cleaning the data should allow researchers to a obtain more up-to-date version of the data, with extra years of longitudinal data added.

CF-EpiNet aims to develop a holistic view of how CF impacts on patients across the life course, and to identify modifiable targets for clinical and policy intervention (e.g. early life exposures, access to therapies, educational support) by the application of modern statistical methods for longitudinal analysis and by direct comparisons with international datasets. Key aims are to optimize the scientific value of the Registry by linking it to data from other administrative datasets, and by undertaking a quality of life survey. The quality of life component of the project has been developed within a portal, ‘My CF Registry’, allowing patient-reported outcome measures to be entered directly by patients. In the future, this portal could enable people with CF to view their historical clinical data, self-report data and opt into additional uses of Registry data that will enhance the value of the Registry to the CF community.

## Strengths and weaknesses of the Registry

Registries can provide valuable insights into variations in clinical outcomes, quality of care and the safety and/or effectiveness of treatments. However, the usefulness and applicability of Registry data rely on the quality of several aspects: the measurements and information recorded; the accuracy of data input, data storage and export; and appropriate data analysis and interpretation, bearing in mind the inherent shortcomings of routinely collected data.^17^^,^^18^

A key strength of the UK CF Registry is the population-level coverage. In the UK, the Registry is estimated to capture almost all of the CF population; any consenting patients attending NHS clinics will have annual data routinely collected into the database. Furthermore, the dataset is of high quality, with robust systems for data cleaning and checking. The UK dataset represents one of the largest national CF datasets outside the USA,^17^ and this provides the statistical power to precisely estimate parameters of interest. The UK CF Registry contains a wide range of clinical, health care and social information, allowing for robust adjustment for appropriate covariates in statistical analyses. A further consequence of the high level of population coverage in the UK, coupled with a universal health care system, is that analyses can cover individuals across the full range of the socioeconomic spectrum in the UK. The unique Registry identification number facilitates longitudinal research, an advantage over other registries which rely on yearly snapshot population data. In addition, the coverage for core variables summarized in the annual reports, such as weight and %FEV_1_, are high.

One key limitation of the UK CF Registry compared with some other CF registries is that it at present mandatorily requires only annual review data, rather than all clinical encounters, which limits some of the research questions that can be addressed. There are also some gaps in information collected, such as primary and secondary non-CF-related care episodes. Data linkage to other national datasets has been a long-standing aim of the UK CF Registry, and the CF-EpiNet project is exploring relevant linkages to include those with primary and secondary care databases, mortality data, census data, databases holding area-level information on environmental exposures and the National Pupil Database. For each of these, linkage poses different ethical and practical challenges and strict data protection guidelines are followed.

Common to many registries, in contrast to inception cohorts, survival times of individuals in the UK dataset are subject to left truncation because the Registry captures the living population at the inception of the Registry and incident cases subsequent to this. This leads to potential survivor bias, whereby the living population at the outset of the Registry represented healthier individuals from their respective birth cohorts who have survived to the point of being included in the dataset. This is a common issue in registry analyses. There are strong cohort effects in the data, which are likely to represent a mixture of survivorship effects, and the ‘true’ cohort effects representing improving treatment over time, as demonstrated in other studies.^7^^,^^19^ With longer follow-up, as the UK CF Registry matures, separating age and cohort effects will become possible and eventually it will be possible to analyse incident individuals alone, ensuring that the longitudinal experience of all individuals from a particular birth cohort will be captured.^18^ Making projections about outcomes for people with CF in the future will always be a challenge, in particular because there have been and continue to be substantial improvements in treatment and care over a relatively short time period.

## Data resource access

All the information in the UK CF Registry is held confidentially and available only to two assigned members of the UK CF Registry data management team. Clinical teams can access only the data that relate to patients in their care, and are required to validate their identity using two-factor authentication before logging into the system. The system also provides an audit trail for any data access. The CF Registry is registered under the Data Protection Act (1998) which was designed to provide a legal framework upon which to protect the privacy of personal data when used with information technology.

Data are stored within secure Microsoft Azure data centres in Ireland and Holland, which are certified as suitable for official government data, including health care data. The database servers are not directly connected to the internet; all administrative access is via a secure VPN connection which requires a dedicated security certificate to allow access. The application hosting environment has been security-tested by certified security consultants. All data are viewed and entered via a secure HTTPS web portal.

NHS Research Ethics approval (Huntingdon Research Ethics Committee 07/Q0104/2) has been granted for the collection of data into the UK CF Registry. Each patient or their parent provided written informed consent for collection of data in the registry as outlined above, and this includes for use of pseudonymized data in research. There is a formal process for requesting access to the UK CF registry, and an application form can be found using this link: [www.cysticfibrosis.org.uk/registry]. In 2016 there were 29 applications for access to anonymized Registry data; 26 were approved after review by the UK CF Registry Research Committee, with the remaining three requests being withdrawn or rejected.

Profile in a nutshell

- The UK Cystic Fibrosis Registry is a national, secure, centralized database sponsored and managed by the Cystic Fibrosis Trust. It was set up to record longitudinal health data on people with cystic fibrosis (CF) in England, Wales, Scotland and Northern Ireland. Containing data on almost all people with CF in the UK, it is one of the largest and most complete national CF databases available.
- First established in 1995, to date the UK CF Registry has captured data on over 12 000 individuals with a combined total of more than 100 000 annual assessments.
- Patient data are recorded in the Registry after a confirmed CF diagnosis and consent for data to be collected has been obtained. Subsequently, health data are added to the Registry at every annual assessment.
- Data are collected in several key areas including: demographics (including genotyping andand diagnosis data), hospital admissions and intravenous therapies, pulmonary function, chronic medications, culture andand microbiology, health complications, nutritional assessment, physiotherapy, lifestyle and outcomes (death and transplants).
- There is a formal process for requesting access to the UK CF Registry. An application form and more details can be found using the following link: [https://www.cysticfibrosis.org.uk/the-work-we-do/uk-cf-registry/apply-for-data-from-the-cf-registry].

## Funding and competing interests

E.G. and R.C. work for the CF Trust; all other authors are part of the CF-EpiNet Strategic Research Centre supported by a grant from the Cystic Fibrosis Trust. S.C. is a principal investigator (PI) for a CF Trust Registry-based pharmacovigilance (PASS) study for Pharmaxis, and is a PI for a CF Trust Vertex-funded Registry-based observational study. D.B. is a principal investigator for two CF Trust Registry-based pharmacovigilance studies for Vertex and Teva pharmaceutical companies. DTR is funded by the MRC on a Clinician Scientist Fellowship (MR/P008577/1).
